# Anti-Melanogenic Effects of *Takifugu flavidus* Muscle Hydrolysate in B16F10 Melanoma Cells and Zebrafish

**DOI:** 10.3390/md22050206

**Published:** 2024-04-29

**Authors:** Jinjin Hu, Bei Chen, Shuaijie Qu, Shuji Liu, Xiaoyu Yang, Kun Qiao, Yongchang Su, Zhihui Liu, Xiaoe Chen, Zhiyu Liu, Qin Wang

**Affiliations:** 1 College of Food and Pharmacy, Zhejiang Ocean University, Zhoushan 316022, China; hujinjin@zjou.edu.cn; 2 Key Laboratory of Cultivation and High-Value Utilization of Marine Organisms in Fujian Province, Fisheries Research Institute of Fujian, Xiamen 361000, China; chenbeifjfri@foxmail.com (B.C.); cute506636@163.com (S.L.); qiaokun@xmu.edu.cn (K.Q.); suyongchang@stu.hqu.edu.cn (Y.S.); 3 School of Life Sciences, Xiamen University, Xiamen 361100, China; qsjqsj1993@sina.com (S.Q.); 21620221153523@stu.xmu.edu.cn (X.Y.); 4 College of Food Sciences & Technology, Shanghai Ocean University, Shanghai 201306, China; 18595493198@163.com

**Keywords:** *Takifugu flavidus*, tyrosinase inhibitor, inhibitory mechanism, computational simulation, skin-whitening

## Abstract

Abnormal melanogenesis can lead to hyperpigmentation. Tyrosinase (TYR), a key rate-limiting enzyme in melanin production, is an important therapeutic target for these disorders. We investigated the TYR inhibitory activity of hydrolysates extracted from the muscle tissue of *Takifugu flavidus* (TFMH). We used computer-aided virtual screening to identify a novel peptide that potently inhibited melanin synthesis, simulated its binding mode to TYR, and evaluated functional efficacy in vitro and in vivo. TFMH inhibited the diphenolase activities of mTYR, reducing TYR substrate binding activity and effectively inhibiting melanin synthesis. TFMH indirectly reduced cAMP response element-binding protein phosphorylation in vitro by downregulating melanocortin 1 receptor expression, thereby inhibiting expression of the microphthalmia-associated transcription factor, further decreasing TYR, tyrosinase related protein 1, and dopachrome tautomerase expression and ultimately impeding melanin synthesis. In zebrafish, TFMH significantly reduced black spot formation. TFMH (200 μg/mL) decreased zebrafish TYR activity by 43% and melanin content by 52%. Molecular dynamics simulations over 100 ns revealed that the FGFRSP (T-6) peptide stably binds mushroom TYR via hydrogen bonds and ionic interactions. T-6 (400 μmol/L) reduced melanin content in B16F10 melanoma cells by 71% and TYR activity by 79%. In zebrafish, T-6 (200 μmol/L) inhibited melanin production by 64%. TFMH and T-6 exhibit good potential for the development of natural skin-whitening cosmetic products.

## 1. Introduction

The generation of melanin and the deposition of skin pigments are the most important endogenous protective factors against skin damage from solar ultraviolet radiation and photocarcinogenic effects [1]. However, excessive synthesis and accumulation of melanin can lead to various skin problems, including melasma, chloasma, periorbital hyperpigmentation, freckles, neurodegeneration associated with Parkinson’s disease, and an increased risk of skin cancer [2,3]. Accordingly, the regulation of melanin production has become a research hotspot. Tyrosinase (TYR) is crucial for melanin formation; thus, the inhibition of TYR activity is a main strategy for reducing melanin production. However, compounds such as kojic acid, hydroquinone, arbutin, and ascorbic acid, which are commonly used in skin-whitening products, can cause harmful side effects and have been banned by some national health commissions. For example, prolonged use of hydroquinone carries the risk of permanent skin damage and exogenous ochronosis [4], whereas the long-term use of kojic acid can damage skin barrier function [5]. Therefore, the identification of safe and effective inhibitors of melanin production is urgently needed.

Bioactive peptides derived from natural products are increasingly being recognized for their diverse metabolic and physiological regulatory functions in the human body. Most demonstrate excellent biocompatibility and high safety, positioning them at the forefront of international research in both the food and cosmetic industries [6]. Recent research has focused on the isolation and purification of such peptides, particularly those demonstrating TYR inhibitory activity from various plant and animal sources [7,8,9]. Owing to extreme environmental conditions, marine organisms can produce biologically active peptides with structures and properties that differ from those of terrestrial organisms. For example, Meng et al. [10] used *Bacillus licheniformis* protease to hydrolyze the pearl oyster *Pinctada martensii*. Subsequent isolation, purification, molecular docking, and bioactivity studies revealed that peptide W3 exhibited significant TYR inhibitory activity and could be used as a TYR inhibitor. Recently, scytonemin, isolated from seaweed and cyanobacterial extracts, was shown to effectively reduce mushroom TYR activity in vitro [11]. Currently, marine whitening peptides are mainly derived from algae and microorganisms, with limited research on peptides from fish.

*Takifugu flavidus* is a fish species farmed in Fujian Province, China, for its delicious taste and high nutritional value. Accordingly, it has been well characterized through studies on its artificial reproduction, nutrient composition, and genome analysis [12,13,14]. In preliminary analyses, we found that *T. flavidus* muscle hydrolysate (TFMH) showed a marked whitening effect on the skin and could serve as a potential source of natural TYR inhibitors. In the present study, we utilized response surface experiments to determine the optimum conditions for enzymatic hydrolysis of *T. flavidus* muscle. Subsequently, in vitro and in vivo experiments were conducted to evaluate the inhibitory effects of the hydrolysates on melanin production. Additionally, we screened peptides from TFMH for predicted melanin inhibitory effects, and we investigated the inhibitory mechanism and confirmed the efficacy of the novel peptide FGFRSP (T-6) using advanced techniques such as nano-HPLC-tandem mass spectrometry (nano-HPLC-MS/MS), virtual screening, and molecular docking.

## 2. Results

### 2.1. Extraction of TFMH

#### 2.1.1. Selection of the Optimal Protease

**Figure 1:**
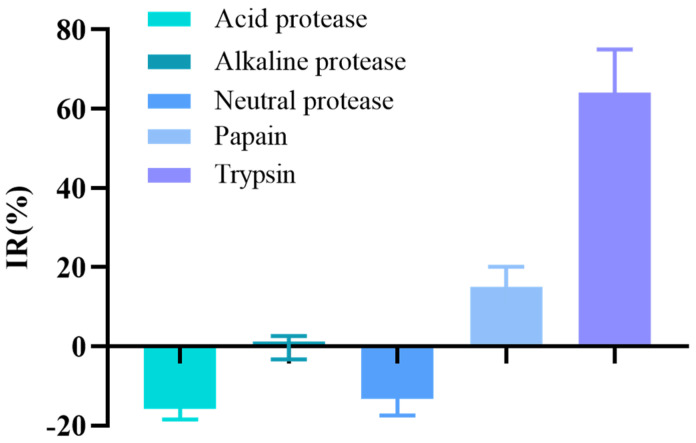
Effects of different proteases on the inhibition rate (IR) of mTYR activity.

Figure 1 shows the results of protease screening. Under specific conditions, different hydrolysates of *T. flavidus* muscle exhibited distinct differences in their mushroom TYR (mTYR) inhibitory activities. Among the tested proteases, trypsin and papain hydrolysates exhibited some degree of mTYR inhibitory activity. In addition, trypsin hydrolysate displayed a significantly greater suppression of mTYR inhibitory activity than that of papain hydrolysate when treated under optimum conditions. In contrast, alkaline, neutral, and acidic hydrolysates did not inhibit mTYR activity. Accordingly, trypsin was used for the subsequent experiments.

#### 2.1.2. Single-Factor Experiments

**Figure 2:**
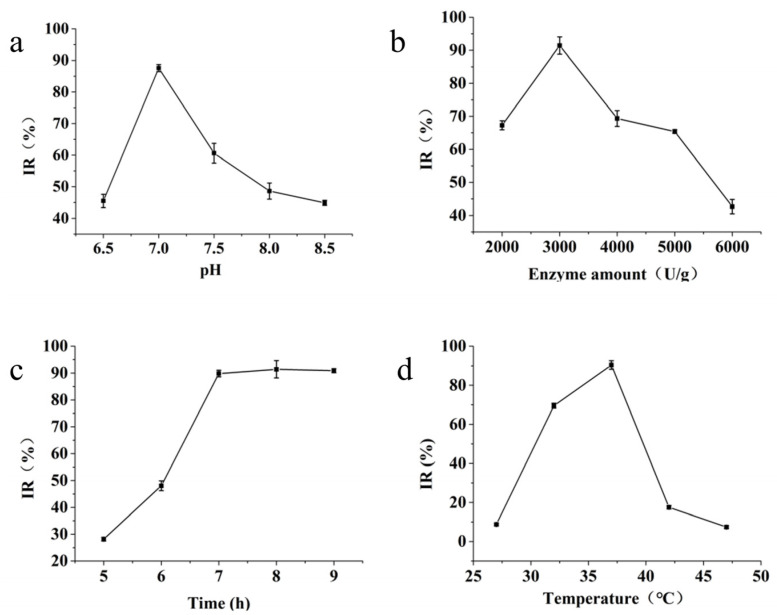
Effects of different factors on the inhibition of mushroom TYR activity. (**a**) Effect of pH on protease activity; (**b**) effect of enzyme amount on protease activity; (**c**) effect of enzymolysis time on protease activity; (**d**) effect of enzymolysis temperature on protease activity.

pH directly affects the activity of enzymes, with both excessively high and excessively low pH levels negatively affecting enzyme activity. As shown in Figure 2a, within the pH range of 6.5–7.0, the inhibition of mTYR activity by the hydrolysate increased with elevated pH. However, within the pH range of 7.0–8.5, the inhibition of mTYR activity by the hydrolysate decreased with increasing pH. Therefore, we speculated that at pH 7.0, the enzymatic hydrolysis reaction exhibits higher trypsin activity, thus establishing pH 7.0 as the ideal condition.

Figure 2b shows that the addition of trypsin from 2000 to 3000 U/g led to enhanced inhibition of mTYR activity in the hydrolysate. However, this inhibition decreased when the enzyme dosage increased from 3000 to 6000 U/g. Therefore, the optimal enzyme concentration was determined to be 3000 U/g.

As shown in Figure 2c, with a gradual extension of the enzymatic digestion duration, the inhibitory capacity of the enzymatic hydrolysate on mTYR activity steadily increased, reaching a stable state after 7 h. To shorten the production cycle, an enzymatic hydrolysis time of 7 h was therefore adopted.

As shown in Figure 2d, within the temperature range of 27 to 37 °C, the inhibition rate of mTYR activity by the hydrolysate increased with the rise in temperature. However, when the temperature increased from 37 to 47 °C, the inhibitory effect of the hydrolysate on mTYR activity diminished.

#### 2.1.3. Model Fitting and Statistical Evaluation

Considering the interaction among various factors and the results based on single-factor studies, an optimization design using response surface methodology (RSM) was conducted for three factors: the amount of enzyme, pH, and temperature. Seventeen experimental runs were conducted using a Box–Behnken design. The response variable for each experiment was the rate of inhibition of the mTYR activity. Table 1 presents the design and results of the response surface test.

In particular, Table 1 lists the results of the regression models developed using multiple quadratic regression for the process parameters. The regression equation was as follows:

IR = 91.10 − 0.88A + 0.06B − 0.69C − 0.49AB + 0.055AC − 1.17BC − 7.85 A^2^ − 6.97 B^2^ − 6.37C^2^,

where A, B, and C represent the temperature, amount of enzyme, and pH, respectively. This equation delineates the interrelationships between these factors and their effect on the response value. The detailed results of the variance analysis are presented in Table 2.

The results indicated that the model showed an extremely significant difference (*p*  < 0.01). Moreover, the non-significant *p*-value (*p* = 0.4199) of the lack of fit indicated that the model fits well and accurately reflects the real experimental conditions. The correlation coefficient R^2^ was 0.9617, indicating that most of the variation in the response could be explained by the regression model equation. In addition, R^2^_Adj _ = 0.9324, indicating that the model could explain 91.24% of the change in the response value. Hence, the model was capable of analyzing and predicting the inhibition of mTYR activity using TFMH. In the analysis of variance (ANOVA) results, the larger the *F*-value, the more prominent the effect of this factor was on the experimental response values. From these results, we determined that the order of influence on mTYR activity was temperature > pH > enzyme dosage. The results of ANOVA of the regression equations highlighted that the quadratic terms A^2^, B^2^, and C^2^ in the model had a highly significant effect on the inhibition rate of mTYR activity (*p* < 0.01).

**Figure 3:**
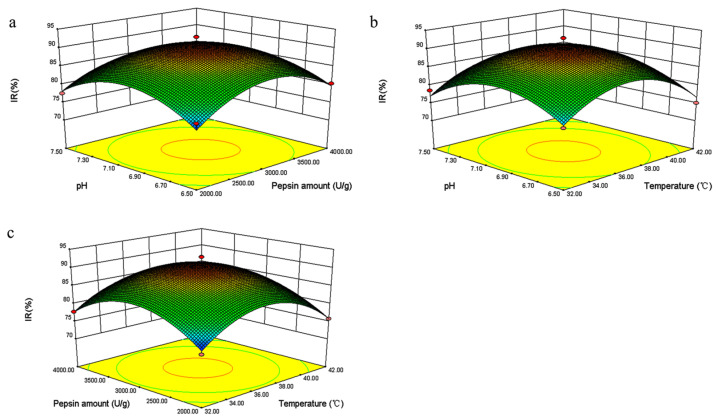
3D surface plots depicting the interactive effects of pH, heating temperature (°C), and pepsin amount (U/g) on the inhibition rate (IR%) of *Takifugu flavidus* muscle hydrolysate (TFMH). (**a**) illustrates the interactive effects between pH and pepsin amount; (**b**) demonstrates the interactive effects of heating temperature and pH; and (**c**) shows the interactive effects of pepsin amount and temperature. The steeper the surface and the denser the contours, the more significant the effect and the stronger the interaction between the two factors.

The analysis indicated that several factors, including pH, the amount of enzyme, and temperature, could significantly influence enzyme activity. The steeper the response surface curve, the more significant the interaction. A response surface diagram (Figure 3) was then constructed according to the multiple nonlinear regression equation. The pH, amount of enzyme, and hydrolysis temperature were confirmed to influence the acquisition of bioactive TFMH as reflected by the steep curve.

#### 2.1.4. Validation of the Model

Based on the models presented above, the optimal parameters for TFMH were identified as follows: added amount of trypsin of 3011 U/g, reaction temperature of 36.9 °C, reaction time of 8 h, hydrolysis at pH 7.0, and liquid-to-material ratio of 1:10. The model predicted an mTYR activity inhibition rate of 91.14%, closely matching the actual observed rate of 90.42%, with relative errors of approximately 0.8%. Therefore, the optimal process conditions obtained through response surface optimization can be practically applied.

#### 2.1.5. Molecular Weight Distribution of TFMH

The molecular weight distribution of TFMH after enzymatic hydrolysis under optimal conditions is presented in Table S1. In TFMH, the proportions of peptides with molecular weights < 1, 1–5, and >5 kDa were 97.31%, 2.68%, and 0.01%, respectively.

### 2.2. Inhibitory Effect of TFMH on TYR Enzyme Activity

**Figure 4:**
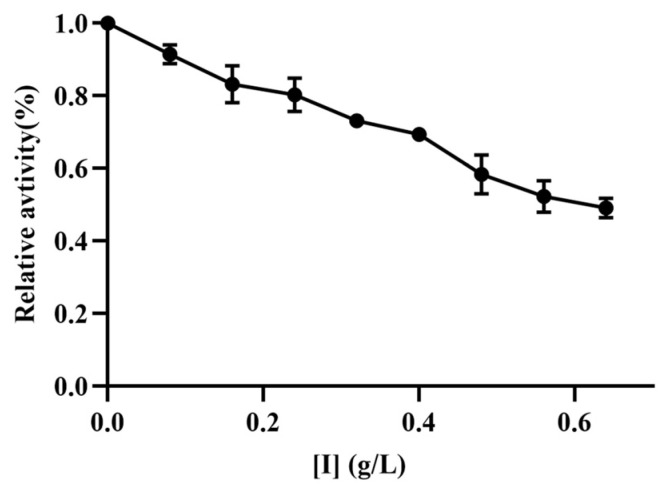
Effects of TFMH on the relative activity of mTYR diphenolase.

TYR is the rate-limiting enzyme in melanogenesis and exerts a significant influence during the conversion of tyrosine into dihydroxyphenylalanine (DOPA) and dopaquinone [15]. In this study, L-tyrosine was used as a substrate to investigate the inhibitory effects of TFMH on the diphenolase activities of mTYR. Concurrently, as the concentration of TFMH increased, a concentration-dependent decrease in enzyme activity was observed, as shown in Figure 4. When the concentration of TFMH reached 0.64 g/L, the remaining bisphenol enzyme activity was 49.15% of that of the control group.

### 2.3. Inhibitory Activity of TFMH on Melanogenesis in B16F10 Melanoma Cells and Mechanism of Action

**Figure 5:**
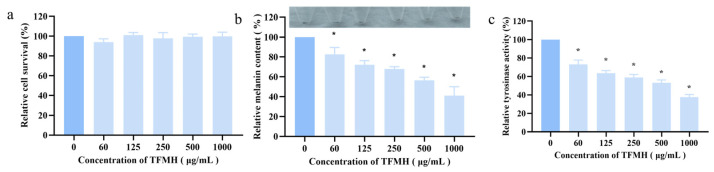
Effects of TFMH on melanin synthesis in B16F10 cells. (**a**) Effects of TFMH on B16F10 cell survival; (**b**) effects of TFMH on B16F10 cell pigmentation; (**c**) effects of TFMH on the TYR activity of B16F10 cells. The data were analyzed by one-way ANOVA followed by Dunnett’s multiple comparison tests. Means with “*” differ signifcantly with the group without TFMH incubation (*p* < 0.05).

As shown in Figure 5a, the results of an MTS assay indicated that TFMH exhibited no toxicity toward B16F10 cells within the concentration range of 0–1000 μg/mL, thus not affecting their growth and proliferation. Further evaluation of melanin content and TYR enzyme activity in B16F10 cells treated with TFMH at concentrations ranging from 0 to 1000 μg/mL revealed a dose-dependent decrease in melanin content (Figure 5b). Additionally, with the increase in TFMH concentration, a significant reduction in TYR enzyme activity was observed, reaching a decrease of 38% at the highest TFMH concentration (Figure 5c).

We also explored the effect of TFMH on the expression of key melanogenesis-related proteins. Treatment with different concentrations of TFMH for 60 h decreased microphthalmia-associated transcription factor (MITF) and TYR protein expression. When the TFMH concentration reached 1000 μg/mL, the expression levels of melanocortin 1 receptor (MC1R), TYR, MITF, tyrosinase related protein 1 (TYRP1), and dopachrome tautomerase (DCT) were all reduced to less than 50% of that of the control group.

**Figure 6:**
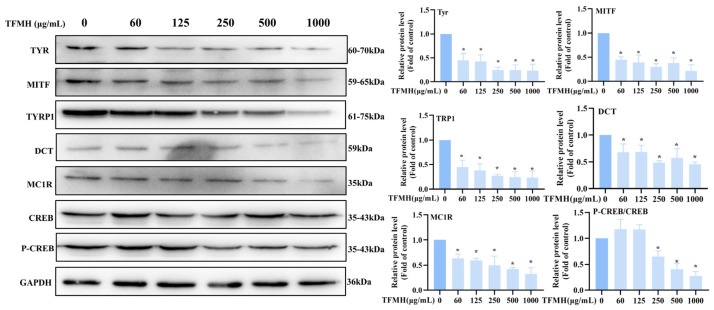
Effects of TFMH on the expression of TYR/MITF/TRP1/DCT/MC1R/CREB proteins in B16F10 cells. The data were analyzed by one-way ANOVA followed by Dunnett’s multiple comparison tests. Means with “*” differ signifcantly with the group without TFMH incubation (*p* < 0.05).

To further explore the inhibitory mechanisms of TFMH in B16F10 cells, we examined the changes in the cAMP response element-binding protein (CREB) pathway after incubating B16F10 cells with TFMH for the indicated periods. As shown in Figure 6, the expression levels of total CREB remained unchanged following TFMH treatment. Conversely, CREB phosphorylation initially increased, followed by a subsequent decrease with increasing TFMH concentrations. When the TFMH concentration reached 1000 μg/mL, the ratio of phosphorylated CREB to total CREB was reduced by 30% compared to that of the control group. Therefore, we speculated that TFMH inhibits melanogenesis in B16F10 cells via the CREB regulatory pathway.

### 2.4. Effect of TFMH on Melanogenesis in Zebrafish

**Figure 7:**
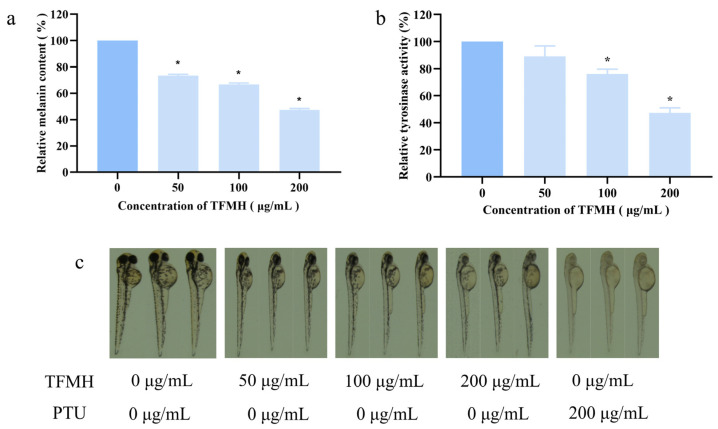
Effect of TFMH on melanin synthesis in zebrafish. (**a**) Effects of TFMH on melanin deposition in zebrafish embryos; (**b**) effects of TFMH on TYR activity in zebrafish embryos; (**c**) effects of TFMH on melanin content in zebrafish embryos. The data were analyzed by one-way ANOVA followed by Dunnett’s comparison tests. Means with “*” differ signifcantly with the group without TFMH incubation (*p* < 0.05).

Zebrafish are frequently used as an animal model for screening melanin inhibitors, especially TYR inhibitors [16,17], as melanogenesis in zebrafish embryos is initiated approximately 24 h post-fertilization (hpf), making them amenable to cultivation and convenient for observation. As we observed that TFMH at concentrations between 0 and 200 μg/mL had no effect on zebrafish survival (Table S2), subsequent experiments were conducted within this concentration range. Zebrafish embryos were cultured in medium containing TFMH for 48 hpf, followed by observation, photography, and video recording. As a positive control, embryos were treated with 0.2 mM propylthiouracil (PTU). Representative results are shown in Figure 7a. In the control group, pigmentation deposition was observed in the eyeball, dorsal region, and yolk sac. Following PTU treatment, melanin deposition in these regions was markedly reduced. In comparison, a gradual decrease in melanin deposition was observed in these regions with increasing TFMH concentration, indicating a concentration-dependent effect. This suggested that TFMH can inhibit melanin deposition to a certain extent in various areas of zebrafish, including the eyeball, dorsal part of the head and trunk, and yolk sac. Figure 7b,c reveal that compared to the results in the control group, TFMH exhibited an inhibitory effect on TYR activity (Figure 7b) and melanin content (Figure 7c) within zebrafish bodies, demonstrating a concentration-dependent inhibition pattern. At the highest concentration of 200 μg/mL, enzyme activity was suppressed by 43% (Figure 7b) and melanin content was reduced by 52% (Figure 7a).

### 2.5. Safety Assessment of TFMH

**Figure 8:**
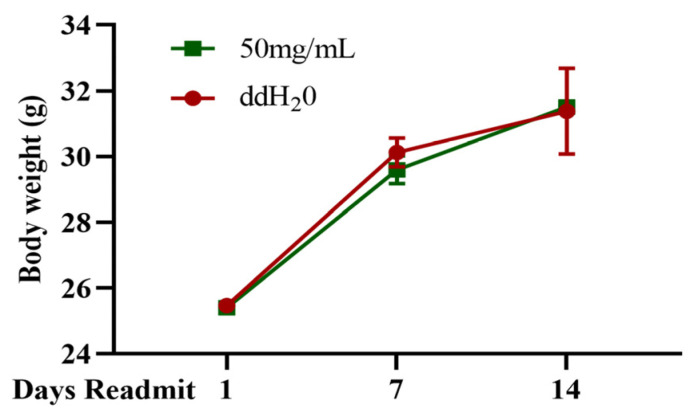
Observation records of in vivo TFMH acute toxicity tests.

According to the detection method outlined in GB 15193.3-2014 [18], mice were orally administered TFMH at a dose of 15 mg/g and continuously observed for 14 days. The results revealed no observable abnormalities in mice in terms of appearance, behavior, mental state, excretion, appetite, fur color, or respiration. No unusual secretions occurred from the eyes, nose, or mouth of the mice. The changes in the body weights of the mice were not significantly different from those in the control group (Figure 8). No deaths were reported, and anatomical examination of the internal organs did not reveal any abnormal lesions. These results suggest that TFMH is not toxic to mice.

### 2.6. Identification and Evaluation of Bioactive Peptides for Melanogenesis Inhibition in TFMH

Nano-HPLC-MS/MS was used to identify the peptide sequences of TFMH. After activity prediction and toxicity assessment, 388 peptide sequences were screened. Virtual screening was then conducted targeting the TYR protein (PDBID: 2Y9X) as the receptor. Strong binding between the protein and compound is reflected in higher absolute values of the molecular docking scores, with the magnitude of the binding strength positively correlating with the absolute value of the score [19]. Fifteen promising peptide sequences were obtained based on Grid Scores and binding patterns, as listed in Table 3.

**Figure 9:**
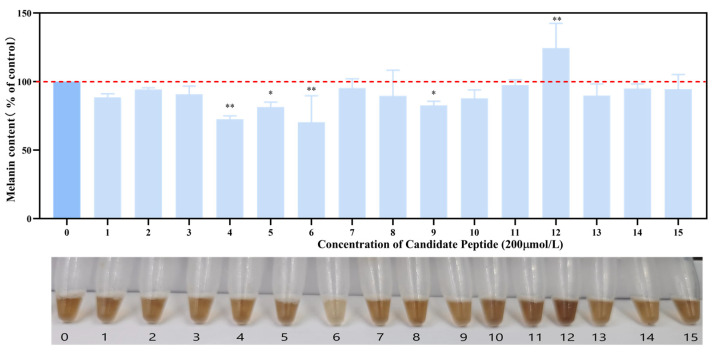
Effects of 15 candidate peptides on melanin synthesis in B16F10 cells. The data were analyzed by one-way ANOVA followed by Dunnett’s multiple comparison tests. Means with “*” differ signifcantly with the group without candidate peptide incubation (* *p* < 0.05; ** *p* < 0.01).

The cytotoxicity of the candidate peptides against B16F10 cells was assessed using an MTS assay (Figure S1). Except for T-15, the remaining candidate peptides exhibited no cytotoxicity toward B16F10 cells at concentrations of 200 and 400 μmol/L. After incubating B16F10 cells with 15 peptides at a concentration of 200 μmol/L, melanin content was measured as depicted in Figure 9. Notably, T-4, T-5, T-6, and T-9 significantly inhibited melanin synthesis, with T-6 exhibiting the most pronounced color change. Therefore, T-6 was selected for use in subsequent experiments.

### 2.7. Analysis of the Interaction between T-6 and TYR

**Figure 10:**
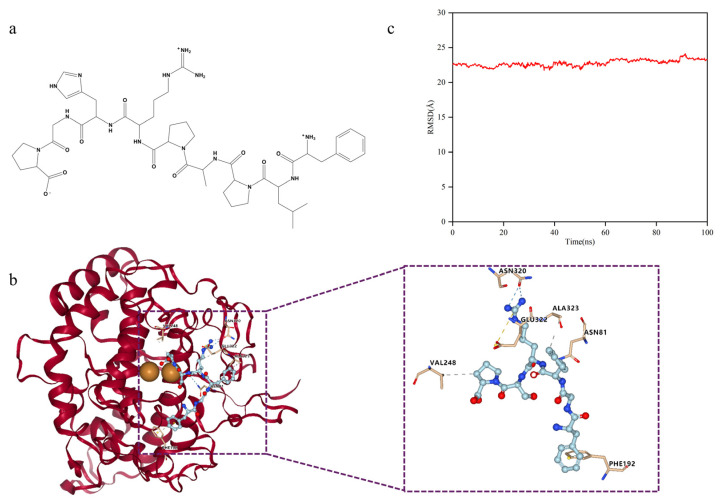
Docking posture analysis of T-6 and 2Y9X. (**a**) Chemical structure of T-6; (**b**) T-6 and 2Y9X docking posture. Detailed interactions between T-6 peptide and mTYR dimer are shown in right, and the residues involved in the interaction with T-6 peptide are presented in stick-ball style. Hydrogen bond forces are indicated by blue dashed lines. Hydrophobic interaction forces are indicated by gray dashed lines. Ionic bond are indicated by orange dashed lines; (**c**) Root mean square deviation analysis of docking complexes.

The active site cavity of TYR is relatively spacious and contains conserved residues such as His244, Glu256, Ala323, Asn320, Phe264, and Tyr140. These sites serve as important targets for the effective screening, optimization, and further development of melanin production inhibitors [20,21]. Docking results for T-6 with 2Y9X are shown in Figure 10b. T-6 binds within the TYR cavity, forming hydrogen bonds with Val248, Ala323, Asn320, and Asn81, establishing an ionic bond with Glu322, and engaging in pi–pi stacking interactions with Phe192. Because T-6 does not directly bind to the binuclear copper ion in this cavity, these interactions among amino acids may play a crucial role in the inhibition of melanogenesis.

We employed molecular dynamics (MD) simulations to investigate the stability of the docking complex. The root mean square deviation (RMSD) is a crucial parameter that indicates the stability of the docking complex [22], reflecting the extent to which the protein molecules deviate from their initial structures during dynamic simulations [23]. MD simulations were conducted using the Amber10 software package [24]. Simulations were initiated with the docking complex of T-6 and 2Y9X as the starting structure.

As shown in Figure 10c, with the passage of time, the change in the RMSD of the docking complex has been maintained within 3Å, and the trend is relatively stable. Therefore, the docking structure of the complex was stable, confirming the reliability of the docking data. However, because of the consistent trend of structural changes over time, we could not determine whether the drastic conformational changes were caused by changes in the protein receptor or in the peptide ligand.

### 2.8. Validation of the Inhibitory Efficacy against Melanin Production

**Figure 11:**
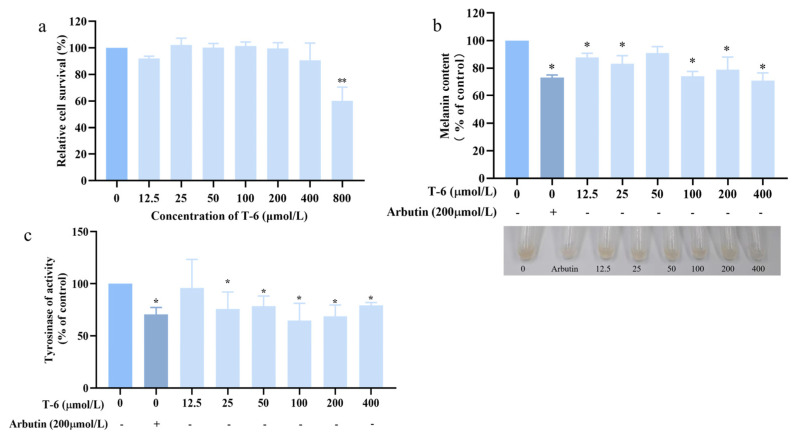
Effects of T-6 on melanin synthesis in B16F10 cells. (**a**) Effect of T-6 on cell activity in B16F10 cells; (**b**) effects of T-6 on melanin content and cell pigmentation in B16F10 cell; (**c**) effects of T-6 on TYR activity of B16F10 cells. The data were analyzed by one-way ANOVA followed by Dunnett’s multiple comparison tests. Means with “*” and “**” differ signifcantly with the group without T-6 incubation (* *p* < 0.05; ** *p* < 0.01).

T-6 cells were co-cultured with B16F10 cells for 60 h, following which cell precipitates were collected and the melanin content within the cells was observed using an alkaline lysis method. The changes in melanin content were determined, with 200 μmol/L arbutin used as the control (Figure 11a). T-6 exhibited an inhibitory effect on melanin content in B16F10 cells, reaching a suppression rate of 71% at a concentration of 400 μmol/L, which was comparable to the melanin inhibition observed with arbutin. As the concentration of T-6 increased, intracellular TYR enzyme activity steadily decreased. At a concentration of 400 μg/mL T-6, TYR activity was reduced to 79%, which was equivalent to that obtained with the positive control arbutin (Figure 11c).

**Figure 12:**
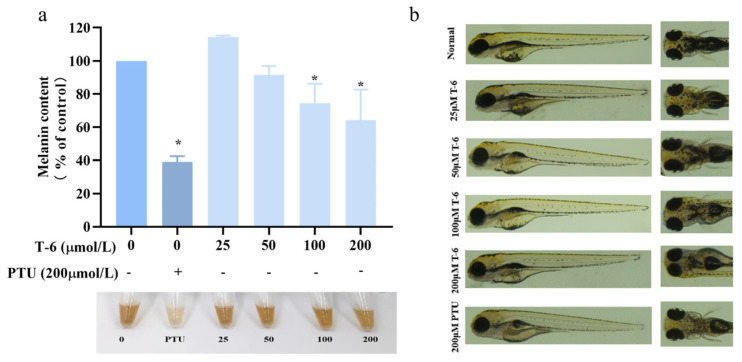
Effect of T-6 on melanin synthesis in zebrafish. (**a**) Effects of T-6 on melanin content in zebrafish embryos; (**b**) effects of T-6 on melanin deposition in zebrafish embryos. The data were analyzed by one-way ANOVA followed by Dunnett’s multiple comparison tests. Means with “*” differ signifcantly with the group without T-6 incubation (*p* < 0.05).

We next investigated the effect of T-6 on melanin formation in zebrafish embryos using PTU as the control treatment, at concentrations of 0, 25, 50, 100, and 200 μmol/L (Table S3). As shown in Figure 12b, melanin production in zebrafish was markedly inhibited compared to that in the control and PTU groups. After drug treatment over 48 hpf, pigment synthesis in the eyes, dorsal region, and yolk sac was visibly reduced, with coloration appearing as scattered, lighter dots. In contrast, the control group exhibited deeper coloration with a dense and patchy pigment distribution. Measurement of melanin content in zebrafish bodies (Figure 12a) indicated that T-6 significantly reduced melanin content and exhibited a concentration-dependent inhibitory effect. At the highest concentration of 200 μmol/L, melanin content was suppressed by 64%.

## 3. Discussion

Hydrolysis of natural proteins using proteases is a proven method for generating peptides that inhibit TYR enzyme activity. Feng et al. [25] produced the TYR-inhibitory tripeptide Phe-Pro-Tyr, by hydrolyzing defatted walnuts using alkaline protease. Han et al. [26] investigated the effects of oyster hydrolysates on melanin synthesis. Karkouch et al. [27] employed pancreatic proteases to hydrolyze and isolate peptides from fava bean protein hydrolysates, which showed both antioxidant properties and TYR inhibition. In turn, our research revealed that the pancreatic protease hydrolysate of *Takifugu flavidus* major muscles exhibited significant mTYR-inhibitory activity. By optimizing the extraction process using single-factor response surface methodology, we achieved an mTYR inhibition rate of up to 90% using TFMH.

TYR inhibition is typically assessed using monophenol substrates, such as tyrosine, or diphenol substrates, such as L-DOPA, to evaluate enzyme activity based on dopaquinone synthesis. In our experiments, TFMH demonstrated diphenol oxidase activities, When the concentration of TFMH reached 0.64 g/L, the remaining bisphenol enzyme activity was 49.15% of that of the control group. Chen et al. [28] observed a similar irreversible binding and inhibition of TYR by HgCl_2_; moreover, Joompang et al. [7] reported irreversible inhibition by TILI-2. We hypothesized that TFMH irreversibly inactivates TYR by reacting with Cu^2+^ at the active site of the enzyme.

Subsequently, we investigated the inhibitory effect of TFMH on melanin production and the underlying cellular mechanisms. Within the non-toxic concentration range for B16F10 cells, TFMH significantly reduced melanin synthesis. Melanin levels depend on TYR expression and activity [29]. In the present study, Western blot analysis showed that TFMH inhibited TYR activity and downregulated TYR expression, resulting in decreased melanin production. Additionally, TFMH markedly suppressed TYRP-1 and TYRP-2 expression. These proteins are regulated by MITF, a key transcription factor that initiates TYR synthesis via the M-box promoter, thereby promoting melanogenesis [30]. We found that TFMH substantially reduced MITF expression. We also explored the role of the cAMP/CREB signaling pathway in TFMH-mediated suppression of melanin synthesis. This pathway, regulated by cAMP and PKA, involves α-MSH binding to the MC1R receptor, leading to ATP conversion to cAMP and subsequent PKA activation. Activated PKA phosphorylates CREB, which then binds to CREB sequences on the MITF promoter, enhancing the expression and transcription of the key melanogenesis regulators, TYR, TRP1, and TRP2 [31]. Our results demonstrated a decrease in MC1R and P-CREB expression within the cells, corresponding to an increase in TFMH concentration. This suggests that TFMH disrupts melanin synthesis via the cAMP/CREB signaling pathway [32]. Specifically, TFMH downregulated MC1R expression, indirectly reducing CREB phosphorylation. Lower levels of CREB phosphorylation lead to suppressed *MITF* gene expression, which in turn decreases TYR, TRP1, and DCT expression, culminating in the inhibition of melanin synthesis. The inhibition or blockage of the cAMP/CREB signaling pathway is recognized as a primary mechanism by which active compounds attenuate melanin production. For example, parallel research has identified various agents, including marine polyphenols [33] and peptides [32], capable of inhibiting melanin production via the cAMP/CREB pathway.

In this study, the receptor 2Y9X was used for molecular docking with the *Chrysophrys* major peptide database, employing Dock for its automated features [34]. Virtual screening and efficacy validation identified T-6 as a significant inhibitor of melanin production. Molecular docking analysis showed that T-6 binds within the TYR cavity, forming hydrogen bonds with Val248, Ala323, Asn320, and Asn81; an ionic bond with Glu322; and engaging in pi–pi stacking interactions with Phe192. The active site of TYR is divided into three distinct regions [35]: a substrate-binding pocket with six histidine residues and a binuclear copper ion; a hydrophobic region comprising Val248, Phe264, Val283, and Pro284; and a solvent-exposed region with Glu189 and Arg268 [36,37,38]. Docking simulations indicated that T-6 may primarily interact with the first and second regions, consequently inhibiting enzyme activity. The ability of T-6 to inhibit TYR activity and melanin production in B16F10 cells was confirmed. At a maximum concentration of 400 μmol/L, the effect of T-6 on melanin content was comparable to that of arbutin, although its inhibitory efficacy was less potent than that of TFMH. The melanin-inhibiting effects of TFMH may be attributable to the synergistic action of multiple peptides with varying properties.

Zebrafish, a widely used vertebrate model organism, shares significant organ systems and genetic sequence similarities with humans [39]. Hsiao et al. [40] developed a zebrafish phenotype-based screening platform to evaluate the whitening properties of Taiwanese plant extracts. In early developmental studies, Agalou et al. [41] identified melanin-inhibitory effects in hawthorn (*Crataegus pycnoloba*), whereas Ouyang et al. [42] examined the influence of Japanese white birch (*Betula platyphylla*) on melanin production using a zebrafish model.

Our study showed that both TFMH and T-6 effectively inhibited melanin accumulation in zebrafish. Pigmentation in the eyes and dorsal regions was lighter and displayed a spotted distribution pattern, as opposed to the darker, uniformly distributed pigment observed in the control group. Typically, zebrafish embryos are cultured in a medium containing PTU, a highly effective sulfur-based TYR inhibitor, to inhibit melanin production [43]. Our findings indicated that both TFMH and T-6 significantly reduced melanin content and TYR activity in zebrafish, with their inhibitory effects intensifying with increasing concentrations. Moreover, these agents effectively prevented pigment deposition without evident side effects. Together, these results suggest that T-6 has potential as a natural peptide with skin-whitening applications.

## 4. Materials and Methods

### 4.1. Preparation of TFMH

The muscles of *T. flavidus* (Zhangpu, Fujian, China) were minced into paste under low-temperature conditions and then subjected to enzymatic hydrolysis using trypsin. After completion of the enzymatic reaction, the protease was inactivated by boiling for 10 min. Following centrifugation, the supernatant was collected and referred to as TFMH.

### 4.2. Optimization of the TFMH Extraction Process

#### 4.2.1. Determination of the mTYR Activity Inhibition Rate by Enzymatic Hydrolysate

The method for determining the inhibition rate of mTYR activity by the enzymatic hydrolysate was based on the procedure described by Rangkadilok et al. [44], with slight modifications. In brief, 90 μL of 0.05 M phosphate buffer, 10 μL of TFMH solution, and 50 μL of 2 mM L-tyrosine solution were added to a 96-well plate and placed in a 37 °C constant temperature incubator for 5 min with shaking. Then, 50 μL of mTYR solution (100 U/mL) was added, and the incubation continued for 30 min with shaking. After the reaction, the absorbance at OD_475_ nm was measured.

The mTYR activity inhibition rate was calculated as follows:

Y (%) = [1 − (A_3_ − A_4_)/(A_1_ − A_2_)] × 100,

where A_1_ comprises 100 μL phosphate buffer, 50 μL L-tyrosine solution, and 50 μL mTYR solution; A_2_ is 150 μL phosphate buffer and 50 μL L-tyrosine solution; A_3_ comprises 90 μL phosphate buffer, 10 μL test sample, 50 μL L-tyrosine solution, and 50 μL mTYR solution; and A_4_ is 140 μL phosphate buffer, 10 μL test sample, and 50 μL L-tyrosine solution. The concentration of the phosphate buffer was 0.05 M, that of L-tyrosine solution was 2 mM, and mTYR was 100 U/mL.

#### 4.2.2. Protease Screening

The mTYR activity inhibition rate (IR%) was used to evaluate the enzymatic hydrolysis effects of different proteases. The conditions for the five proteases were set at an enzyme concentration of 5000 U/g, material-to-liquor ratio of 1:10, and reaction time of 8 h. The optimal pH and temperature values for the five proteases are listed in Table 4. After the reaction, boiled enzymatic hydrolysate was used to deactivate the protease. After cooling, the supernatant was collected and the inhibition rate of the enzymatic hydrolysate on mTYR activity was measured. The experiment was repeated thrice.

#### 4.2.3. Single-Factor Experiments for Enzymatic Extraction of TFMH

Using the mTYR activity inhibition rate (IR%) as the experimental indicator, the effects of pH, reaction time, reaction temperature, and enzyme concentration on the mTYR activity inhibition rate of TFMH were investigated. The experiment was repeated thrice.

#### 4.2.4. Optimization of the Enzymatic Extraction Process for TFMH by Response Surface Methodology

Based on the above results, three factors with significant effects on the inhibition rate, namely temperature, enzyme amount, and pH, were selected for investigation using the Box–Behnken design [45]. The mTYR activity inhibition rate (IR%) was set as the experimental indicator with a fixed enzymatic hydrolysis time of 8 h and a liquid-to-material ratio of 1:10 (g/mL). Table 5 lists the levels of these factors.

#### 4.2.5. Molecular Weight Distribution of TFMH

The proportion of protein hydrolysates with a relative molecular mass of <1 kDa was determined using a high-efficiency gel chromatography filtration method, referencing GB/T22729-2008 [46].

### 4.3. Inhibition Effect of TFMH on mTYR Diphenolase Activity

Using a method described previously [47], the inhibitory effect of TFMH on mTYR diphenolase activity was examined. Relative enzyme activity was determined by measuring the optical density (OD) at 475 nm in a reaction system with varying concentrations of TFMH.

### 4.4. Cell Culturing

Mouse B16F10 cells were donated by the School of Life Sciences, Xiamen University, and cultured in Dulbecco’s modified Eagle medium (C11995500BT, Gibco, Gaithersburg, MD, USA) containing 10% fetal bovine serum (F8687, Merck, Darmstadt, Germany) and 1% penicillin-streptomycin (C0222, Beyotime Biotechnology, Shanghai, China).

### 4.5. Cell Viability Assay

The effects of TFMH and T-6 on the proliferation of B16F10 cells were determined using the CellTiter 96 AQueous Non-Radioactive Cell Proliferation Assay Kit (Promega, Madison, WI, USA).

### 4.6. Melanin Content and TYR Activity Measurement in B16F10 Cells

Following a previously described method [48] with modifications, cells were seeded at a density of 1.0 × 10^5^ colony forming units (CFUs)/mL in a 6-well plate and cultured for 8–12 h. After incubation for 60 h with different concentrations, the culture medium was removed and the residual medium was washed off with 1 mL of Dulbecco’s phosphate-buffered saline (DPBS) (C14190500BT, Gibco, Gaithersburg, MD, USA) per well. After washing twice, 1.5 mL DPBS was added and the cells were collected using a cell scraper. After centrifugation at 2000 rpm for 15 min, cell pellets were obtained. Then, 150 μL of 1 mol/L NaOH solution containing 10% dimethylsulfoxide (67685, MP Biomedicals, Santa Ana, CA, USA) was added, and the solution was heated at 95 °C for 1 h with vortexing every 10 min to dissolve the melanin completely. After cooling, the solution was transferred to a 96-well plate and the absorbance at OD_405_ nm was measured using a microplate reader to determine melanin content.

For TYR activity measurement, after collecting the cell pellet, 200 μL DPBS buffer containing 1% Triton X-100 (T8200, Solarbio, Beijing, China) and 1 mM phenylmethylsulfonyl fluoride (PMSF) (P0100, Solarbio) was added. After three cycles of freezing and thawing at −80 °C, centrifugation at 12,000 rpm at 4 °C was performed to collect the supernatant for TYR activity determination. The protein concentration in the supernatant was determined using a bicinchoninic acid (BCA) protein assay kit (23227, Thermo Scientific, Waltham, MA, USA) and adjusted to the same level. Then, 50 μL of the supernatant was mixed with 150 μL of 1 mg/mL L-DOPA solution. After a 30 min light-protected reaction at 37 °C, the absorbance at OD_475_ nm was measured using a microplate reader.

### 4.7. Western Blot Analysis for Detection of Cell Melanin Signal Pathway Protein Expression

Cells were cultured following a previously described method [49] with modifications. After washing the residual culture medium with DPBS, the cells were collected in DPBS buffer and lysed in RIPA buffer (high) (R0010, Solarbio, Beijing, China) on ice for 1 h, and the cell suspension was centrifuged at 4 °C for 30 min. The protein concentration in the cell lysate was determined using the BCA method, adjusted to be the same across samples, and equivalent amounts were added to sodium dodecyl sulfate-polyacrylamide gel electrophoresis (SDS-PAGE) Loading Buffer (P1016, Solarbio). After heating at 95 °C for 5 min in a metal bath, the supernatant was collected by centrifugation at 12,000 rpm for 5 min.

Proteins were separated using SDS-PAGE, transferred to a polyvinylidene fluoride membrane, and blocked in a blocking solution (Tris pH 0.1%, Tween 5%, bovine serum albumin) for 2 h at room temperature. After washing the membranes with TBST, they were incubated overnight at 4 °C with antibodies against TYR (A16993, Abclonal, Cambridge, MA, USA), MITF (13092-1-AP, Proteintech, Rosemont, IL, USA), TRP-1 (ab235447, Abcam, Cambridge, UK), DCT (13095-1-AP, Proteintech, Rosemont, IL, USA), MC1R (ab125031, Abcam), CREB (A10826, Abclonal, Wuhan, China), p-CREB (AP0091, Abclonal, Wuhan, China), and GAPDH (CL594-6004, Abclonal, Wuhan, China). After three washes with TBST, the membranes were incubated with secondary antibodies (N20915, N21009, TransGen, Beijing, China) and conjugated with horseradish peroxidase for 1 h. After final washes with TBST, protein visualization was performed using the ECL protein imprinting test reagent (K-12045-D50, Advansta, San Jose, CA, USA). Signal scanning and density analyses were conducted using ImageJ 1.8.0-265 (64-bit) software.

### 4.8. Determination of Zebrafish Embryo Survival Rate

Zebrafish embryos (Xiamen University, Xiamen, Fujian, China) collected 24 h post-fertilization were cultured in a constant-temperature incubator at 28 °C. Zebrafish embryo culture media containing different concentrations of TFMH were prepared. A 6-well plate was used as the culture container, with 25 zebrafish embryos per well. After culture for 72 h, the dead embryos were numbered, and their developmental status was recorded.

### 4.9. Zebrafish Embryo Exposure

Zebrafish embryo culture media with different concentrations were prepared, with 0.2 mM PTU treatment used as a positive control. A 6-well plate containing 25 zebrafish embryos per well was used for culture. After 48 h of incubation, the embryos were observed and photographed using a stereomicroscope.

### 4.10. Determination of Melanin Content and TYR Activity in Zebrafish Embryos

Zebrafish embryos were cultured and treated with drugs as described above. After 48 h of zebrafish embryo culture, centrifugation was performed at 2000 rpm for 5 min. The pellet was washed with ddH_2_O, resuspended in 0.2 mL of 1 M NaOH solution, and dissolved in a metal bath at 95 °C for 0.5 h. The absorbance at OD_405_ nm was measured after cooling.

The collected zebrafish embryos were treated with 1% Triton X-100 solution containing 1 mM PMSF by ultrasonication in an ice bath for 5 min. After centrifugation at 4 °C for 0.5 h (10,000 rpm), the supernatant was collected. The BCA method was used to adjust the protein concentrations. Then, 0.05 mL of the supernatant was added to 0.15 mL of L-DOPA (0.05 mM), thoroughly mixed, and incubated at 37 °C in the dark for 0.5 h. The absorbance at OD_475_ nm was measured.

### 4.11. Mouse Acute Toxicity Test

According to the testing basis of GB 15193.3-2014, TFMH was administered to the mice (SCXK2017-0005, Slaccas, Shanghai, China) by gavage at a dose of 15 g/kg. Each group consisted of 10 male and 10 female mice housed separately in ICR cages. After one week of acclimatization, the mice were subjected to gavage. Mice were fasted for 6 h before gavage but had unrestricted access to water. TFMH was dissolved in ddH_2_O; as a control, mice were administered the same volume of ddH_2_O by gavage. Continuous observations were performed for 14 days, and the organs were observed.

### 4.12. Nano-HPLC-MS/MS Identification of Peptide Sequences

Peptide fractions with melanin-inhibitory effects were subjected to LC-MS/MS analysis using a Q-Exactive Plus mass spectrometer (Thermo Fisher, Waltham, MA, USA) coupled with an EASYY-nanoLC 1200 system. A total of 3 μL was injected (75 μm × 25 cm, Thermo Fisher, Waltham, MA, USA), and the sample was separated over 60 min. The column flow rate was maintained at 300 nL/min, column temperature at 40 °C, and electrospray voltage at 2 kV. The gradient started at 2% B phase, increased nonlinearly to 35% within 47 min, increased to 100% within 1 min, and was maintained for 12 min. The mass spectrometer was operated in the data-dependent acquisition mode and automatically switched between MS and MS/MS. The MS1 parameters were set as follows: scan range, 200–1800 m/s; resolution, 70,000; maximum injection time, 50 ms; and AGC target, 3 × 10^6^. The MS2 high-energy collisional dissociation MS/MS parameters were set as follows: resolution, 17,500; collision energy, 28; maximum injection time, 45 ms; AGC target, 1 × 10^5^; and dynamic exclusion time, 30 s. The mass spectral raw files were analyzed using Peaks Studio version 10.6 (Bioinformatics Solutions Inc., Waterloo, ON, Canada).

### 4.13. Virtual Screening of Peptides

Based on the MS analysis results, peptides with a confidence −log *p* > 20 and molecular weight < 1 kDa were selected. Peptides with bioactivity > 0.75 were preliminarily screened using the Peptide Ranker program (http://distilldeep.ucd.ie/PeptideRanker/ (accessed on 22 April 2024)). A high-activity peptide database was established in Discovery Studio, and toxicity predictions, such as skin carcinogenicity, skin irritation, carcinogenicity, mutagenicity, and eye irritation, were performed using the toxicity module. The crystal structure of mTYR (PDBID: 2Y9X) was obtained from the PDB RCSB database. DOCK 6.9 was conducted to study the binding of high-activity peptides to mTYR, with the peptides selected based on Grid Score values and binding modes.

### 4.14. Data Processing

GraphPad Prism 8.0.2 statistical software (La Jolla, CA, USA) was used for one-way ANOVA to analyze the significant differences in each group of data. *p* < 0.05 was considered statistically significant.

## Figures and Tables

**Table 1:** Box–Behnken design and experimental results.

Run	A	B	C	Inhibition Rate
	Temperature	Enzyme Amount	pH	(IR/%)
1	0	1	−1	80.31
2	−1	−1	0	75.69
3	−1	0	1	78.58
4	0	0	0	92.96
5	0	0	0	92.88
6	1	0	−1	75.06
7	0	0	0	88.21
8	−1	1	0	77.76
9	0	0	0	90.66
10	−1	0	−1	77.81
11	0	−1	−1	78.84
12	0	−1	1	77.54
13	1	−1	0	75.78
14	0	0	0	90.78
15	0	1	1	74.34
16	1	1	0	75.88
17	1	0	1	76.05

**Table 2:** Variance analysis of the regression model.

Source of Variation	Sum of Squares	Degrees of Freedom	Variance	*F*-Value	*p*-Value	Significance
Model	725.41	9	80.60	19.53	0.0004	**
A	6.25	1	6.25	1.51	0.2583	
B	0.024	1	0.024	5.863 × 10^−3^	0.9411	
C	3.80	1	3.80	0.92	0.3696	
AB	0.97	1	0.97	0.24	0.6426	
AC	0.012	1	0.012	2.932 × 10^−3^	0.9583	
BC	5.45	1	5.45	1.32	0.2882	
A^2^	259.56	1	259.56	62.89	<0.0001	**
B^2^	204.49	1	204.49	49.54	0.0002	**
C^2^	170.93	1	170.93	41.41	0.0004	**
Residual	28.89	7	4.13			
Lack of Fit	13.62	3	4.54	1.19	0.4199	
Pure Error	15.28	4	3.82			
Total	754.30	16				
R^2^ = 96.17%, R^2^_Adj_ = 91.24						

** (*p* < 0.01).

**Table 3:** Peptide sequences and docking scores of peptides and TYR.

Sequence Number	Peptide Sequence	Grid Score (kcal/mol)
T-1	SGFPRHR	−153.2497
T-2	LSGFPRHR	−153.2468
T-3	IRWR	−132.6575
T-4	ARWNPAPGP	−126.1295
T-5	WGPDPR	−125.9351
T-6	FGFRSP	−118.9725
T-7	DWPDGRG	−113.1157
T-8	MGRWL	−111.5497
T-9	FFRI	−110.2282
T-10	FMRF	−109.5137
T-11	LWDR	−104.4215
T-12	YPRF	−103.6229
T-13	FIRF	−102.5375
T-14	FNRTPIGW	−126.8967
T-15	IRFR	−128.7539

**Table 4:** Optimal pH and temperature of different proteases.

Number	Protease Species	Optimal pH	Optimal Temperature (°C)
1	Trypsin	7.5	37
2	Acid Protease	4	50
3	Neutral Protease	6	50
4	Papain	6.5	60
5	Alkaline Protease	8.5	55

**Table 5:** Orthogonal factors and levels of the Box–Behnken design.

Level	Factor		
	Temperature/°C (A)	Enzyme Amount/(U/g) (B)	pH (C)
−1	32	2000	6.5
0	37	3000	7
1	42	4000	7.5

## Data Availability

Data obtained in this study are available from the corresponding author upon request.

## Supplementary Materials

The following supporting information can be downloaded at https://www.mdpi.com/article/10.3390/md22050206/s1, Table S1: Molecular weight distribution of TFMH; Table S2: Effects of TFMH on zebrafish growth; Table S3: Effects of T-6 (FGFRSP) on zebrafish growth; Figure S1: Effect of 15 candidate peptides on the viability of B16F10 cells.
