# Supplementary material for: Anti-Melanogenic Effects of Takifugu flavidus Muscle Hydrolysate in B16F10 Melanoma Cells and Zebrafish
Source: Mar Drugs. 2024 Apr 29;22(5):206. doi: 10.3390/md22050206 (PMC11122720; doi:10.3390/md22050206)
Supplement: Supplementary file 1 [file marinedrugs-22-00206-s001.zip › marinedrugs-2961120-supplementary.pdf]

Table S1. Molecular weight distribution of TFMH.

Molecular weight range（kDa）	Proportion of components（%）
>5	0.01
1-5	2.68
0-1	97.31

Table S2. Effects of TFMH on zebrafish growth.

	TFMH Concentration（mg/mL）	Death rate（%）
Control	0	100%
PTU	200	100%
TFMH	50	98.2%
	100	99.0%
	200	95.6%
	400	85.6%

Table S3. Effects of T-6 (FGFRSP) on zebrafish growth.

	T-6 Concentration（μmol/L）	Death rate（%）
Control	0	100%
PTU	200	100%
T-6	25	100%
	50	100%
	100	100%
	200	95.6%
	500	58.7%

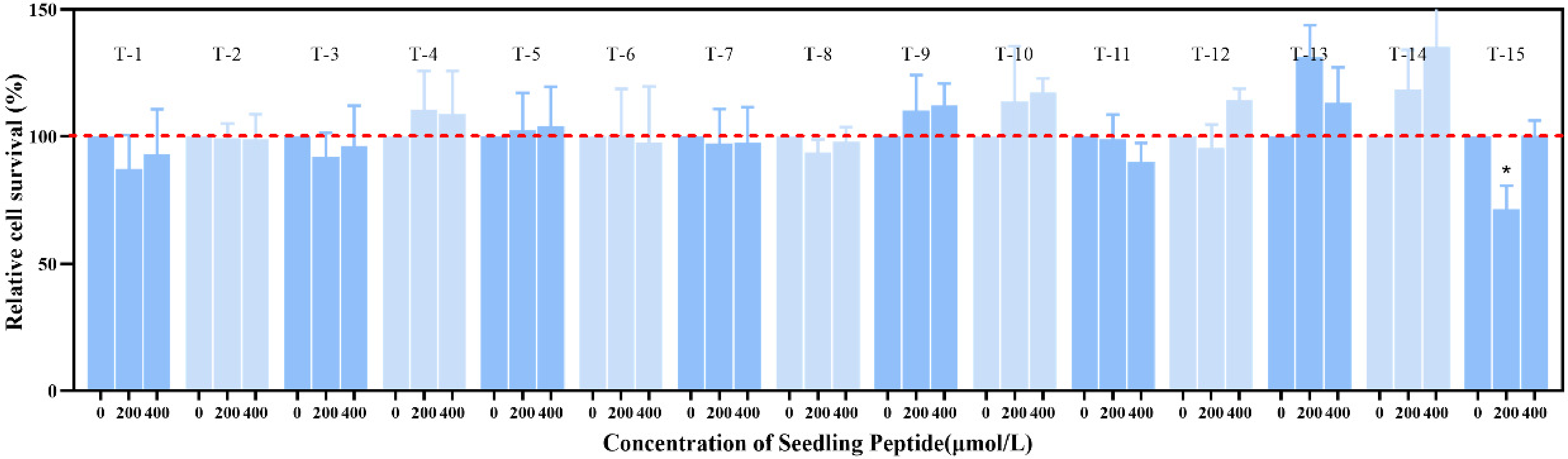

Figure S1. Effect of 15 candidate peptides on the viability of B16F10 cells.
